# Increasing Frequency of Secondary Dengue Infections in Sequential Outbreaks (2016–2024)—Clinical Impact and Diagnostic Challenges

**DOI:** 10.3390/v18070785

**Published:** 2026-07-18

**Authors:** Sonia L. Espindola, Matías Javier Pereson, José Martín Lema, Analía Kachuk, Graciela M. Carballo, Natalia Aloisi, María Noel Badano, Marcos Miretti, Federico A. Di Lello, Patricia Baré

**Affiliations:** 1 Laboratorio GIGA, Instituto de Biología Subtropical (IBS), Consejo Nacional de Investigaciones Científicas y Técnicas (CONICET), Facultad de Ciencias Exactas Químicas y Naturales, Universidad Nacional de Misiones (UNaM), Posadas 3300, Argentina; sonialespindola@gmail.com (S.L.E.); kachuk.analia@gmail.com (A.K.); mmiretti03@yahoo.co.uk (M.M.); 2 Laboratorios CEBAC SRL, Posadas 3300, Argentina; gachicar@gmail.com; 3 Instituto de Medicina Experimental (IMEX), Consejo Nacional de Investigaciones Científicas y Técnicas (CONICET), Academia Nacional de Medicina, Buenos Aires 1425, Argentina; 4 Instituto de Investigaciones Hematológicas (IIHEMA), Academia Nacional de Medicina, Buenos Aires 1425, Argentina; martinlema123@gmail.com (J.M.L.); naloisi@hematologia.anm.edu.ar (N.A.)

**Keywords:** dengue virus, secondary infection, severity, serotype

## Abstract

Successive dengue virus (DENV) outbreaks progressively reshape population immunity, influencing disease expression and diagnostic performance. This study evaluated the impact of secondary infections across sequential outbreaks on disease severity markers, serotype dynamics, and the agreement between routinely used direct diagnostic methods. This retrospective study analyzed 976 serum samples from three outbreaks in Misiones, Argentina. Clinical and serotype analyses included 869 acute-phase dengue cases confirmed by at least one direct detection method (2016: *n* = 512; 2019: *n* = 148; 2024: *n* = 209). A subset of 318 samples, including 107 RT-PCR/NS1-negative samples, was used to compare NS1 antigen rapid diagnostic tests (NS1 Ag) and RT-PCR. Viral serotypes and clinical and laboratory severity markers were evaluated. Secondary infections increased from 31.1% in 2016 to 43.2% in 2019 and 53.1% in 2024 (*p* < 0.001). Serotype distribution shifted from DENV-1 predominance in 2016 (95.1%) to DENV-1/DENV-4 co-circulation in 2019 (60.7%/39.3%) and DENV-2 predominance in 2024 (97.6%). Secondary infections were associated with increased frequency of laboratory severity markers, particularly in 2024, including higher hematocrit, liver enzyme levels and lower leukocyte and platelet counts. Concordance between NS1 Ag and RT-PCR was lower in secondary infections (κ = 0.457 vs. 0.759, *p* = 0.0013). The increasing frequency of secondary infections may influence laboratory markers associated with dengue severity and reduce the diagnostic concordance between direct detection methods, highlighting the need for optimized surveillance and diagnostic strategies during dengue outbreaks.

## 1. Introduction

Dengue fever is a systemic mosquito-borne viral disease caused by dengue virus (DENV), primarily transmitted by *Aedes aegypti*. It has become the most widespread arboviral infection globally, with an estimated 390 million infections annually [1]. In 2024, the Americas reported an unprecedented surge exceeding 14 million cases and more than 12,000 deaths [2]. According to historical records, the province of Misiones in Argentina has experienced three successive dengue outbreaks, since 2016 [3,4,5,6,7,8]. However, how the progressive accumulation of secondary DENV infections across successive outbreaks influences clinical presentation and the performance of routine diagnostic methods has not been fully characterized.

Secondary infections are a key driver of disease burden. The clinical outcome of dengue infection is shaped by serotype prevalence, co-circulation patterns, and the host immune profile, including previous DENV exposure and pre-existing antibody levels, which may confer protection or promote antibody-dependent enhancement [9,10,11,12]. Although secondary heterologous DENV infections are widely recognized as a major risk factor for severe dengue, disease severity is multifactorial and is influenced by viral, host, and immunological factors. Moreover, recent clinical studies have shown that severe dengue may also occur during primary infections, indicating that immune status alone does not fully explain clinical outcome [13,14,15].

Several laboratory parameters reflect disease progression and have been associated with dengue severity. Elevated hematocrit indicates plasma leakage; thrombocytopenia and leukopenia are associated with progression to hemorrhagic shock and increased hepatic enzymes suggest organ involvement and poorer prognosis. Together, these laboratory abnormalities are commonly associated with more severe clinical disease and may assist in patient risk stratification.

During outbreaks, rapid diagnostic tests (RDTs), typically immunochromatographic assays detecting NS1 antigen (NS1 Ag) and/or IgM/IgG, are widely used because they require minimal infrastructure and provide quick results [16,17,18]. During the acute phase, guidelines recommend combining antigen detection, serology, and RT-PCR (when available), interpreted according to illness timing and clinical presentation [18]. However, the timing of sample collection, infecting serotype, viral load, and host immune status influences the performance of direct diagnostic methods. Consequently, the concordance between NS1 antigen detection and RT-PCR may vary according to the immunological profile of the affected population and particularly during large outbreaks.

This study investigated how sequential dengue outbreaks reshape population immunity and how these changes influence laboratory markers associated with dengue severity, serotype dynamics, and the concordance of routine direct diagnostic methods.

## 2. Materials and Methods

### 2.1. Samples and Study Design

This retrospective study was conducted on 976 serum samples collected within the first week after symptom onset from patients with clinically suspected dengue during three sequential outbreaks (2016–2024) in Posadas, Misiones (northeastern Argentina). All samples were referred for laboratory diagnosis. Detailed clinical symptom data were not systematically available; therefore, clinical analyses were limited to the demographic and laboratory variables available for all patients. Acute-phase dengue infection was confirmed by direct detection methods (RT-PCR and/or NS1 Ag), consistent with WHO/PAHO recommendations for the diagnosis of acute dengue during the early phase of illness.

The analysis of serotyping and clinical manifestations included 869 acute-phase dengue cases confirmed by direct detection methods from 2016, 2019, and 2024. Routine clinical and laboratory data were retrieved from the laboratory system and medical records.

To evaluate the agreement between NS1 Ag detection and RT-PCR under different serological conditions, a subgroup of 318 consecutive serum samples was selected. All samples were tested by both direct detection methods and for the presence of IgM and IgG ELISA antibodies. Among them, 211 were positive by at least one direct detection assay. A group of 107 samples negative by both RT-PCR, and NS1 antigen detection was included, representing the range of serological profiles encountered during routine dengue diagnosis. Only samples from 2016 were included to minimize serotype-related differences in antigen detection, when DENV-1 accounted for nearly all circulating strains in the region.

### 2.2. Dengue Diagnostic Tests and Serotyping

Acute dengue infection was confirmed by detection of DENV NS1 antigen, DENV RNA, or both. Dengue virus-specific NS1 antigen (NS1 Ag) was detected using RDT, SD BIOLINE Dengue DUO kit (Demgue NS1 Ag and IgM/IgG, Abbott Diagnostics Korea Inc., Yongin-si, Republic of Korea). Results were interpreted according to the manufacturer’s instructions. Appearance of both the control line and NS1 test line was considered positive.

Viral RNA was detected using the RealStar^®^ Dengue RT-PCR Kit 3.0 (Altona Diagnostics, Hamburg, Germany), a one-step real-time RT-PCR assay designed for the qualitative detection of all four dengue virus serotypes (DENV-1-4). Each RT-PCR run included the manufacturer’s positive, negative, and internal controls, samples exhibiting an amplification signal with a cycle threshold (Ct) < 40 were defined as positive. DENV serotypes were determined using the CDC DENV 1–4 real time PCR assay modified by Santiago et al. [19].

Anti-DENV IgM and IgG antibodies were detected using commercial ELISA kits (DIA.PRO Diagnostic Bioprobes Srl, Milan, Italy) according to the manufacturer’s instructions. Both assays use microplates coated with highly purified immunodominant NS1 antigens representing the four DENV serotypes. For both ELISA assays, samples displaying a sample-to-cut off optical density (OD) ratio (S/Co) greater than 1.1 were considered positive. Both assays report sensitivity and specificity above 98%.

### 2.3. Classification of the Type of Infection

For the 869 acute-phase dengue cases, confirmed by direct detection methods (RT-PCR and/or NS1 Ag), anti-DENV IgG antibodies were used to classify infections as primary or secondary. Because all samples were collected within the first week after symptom onset, detectable anti-DENV IgG was interpreted as evidence of pre-existing immunity since IgG antibodies are usually not detectable during the first week of illness for primary infection. Among the subset of 107 RT-PCR/NS1-negative samples, used exclusively for the diagnostic concordance analysis, IgM/IgG ratios and IgG titers were calculated to distinguish primary from secondary infections. Classification of primary and secondary dengue infections using IgG/IgM or IgM/IgG ratios has been widely validated in ELISA-based studies. Samples with low IgM/IgG rates (<1.7) were considered as secondary infections, consistent with the differential kinetics of the humoral response [20,21].

### 2.4. Routine Laboratory and Biochemical Assays

Routine laboratory analyses were performed for all participants. White blood cell (WBC) and platelet counts were measured using a CELL-DYN Ruby Hematology Analyzer (Abbott Diagnostics). Serum alanine aminotransferase (ALT), aspartate aminotransferase (AST), total protein, albumin, and bilirubin levels were determined by photometric methods using the ALINITY Ci-Series system (Abbott Diagnostics). Laboratory parameters were categorized according to thresholds previously associated with dengue severity [22,23]. Leukopenia was defined as a WBC count < 5000/μL, thrombocytopenia as a platelet count < 100,000/μL, and elevated liver enzymes as ALT or AST levels exceeding the upper limit of the normal reference range (40 U/L).

### 2.5. Statistical Analysis

Continuous variables were compared using Student’s *t*-test or the Mann–Whitney U test depending on data distribution. Categorical variables were analyzed with Chi-square or Fisher’s exact test. To evaluate the association between age and disease severity, age was analyzed both as a continuous variable and as a categorical variable after stratification into four groups: 1–18, 19–30, 31–45, and 46–60 years. Confidence intervals were set at 95% (CI95); *p* value < 0.05 was considered statistically significant. Concordance between NS1 Ag-RDT and RT-PCR was assessed using Cohen’s kappa coefficient (κ). Agreement was classified as fair (κ < 0.40), moderate (κ = 0.41–0.60), high (κ = 0.61–0.80) and near perfect (κ > 0.80). Kappa coefficients were compared using approximate Z-tests based on their standard errors. Statistical analyses were performed using GraphPad Prism 11.0.2 (92) software. Artificial intelligence (AI)-based tools (ChatGPT, OpenAI, based on the GPT-5.5 large language model) were used to assist in Kappa exploratory statistical comparisons. All statistical approaches, interpretations, and conclusions were independently reviewed and validated by the authors.

### 2.6. Ethical Aspects

Protocols and procedures were approved by the Ethical Committee of the Academia Nacional de Medicina of Buenos Aires (CEIANM) (TI 12443/16/X; 88/26/CEIANM) and the Ethical Committee of the “Investigación Provincial”, Misiones (CEIP). Written informed consent was obtained from all participants for the use of samples and clinical data in research. Data were stored in a secure, coded database accessible only to study investigators, with personal identifiers replaced by alphanumeric codes to ensure confidentiality.

## 3. Results

### 3.1. Characteristics of the Study Population

A total of 869 patients with acute dengue confirmed by direct detection methods were included in this study, comprising 512 patients from the 2016 outbreak, 148 from 2019, and 209 from 2024. The proportion of female patients was similar across the three outbreaks. In 2016, females represented 57.2% (293/512); in 2019, 53.4% (79/148); and in 2024, 56.9% (119/209). Overall, no significant differences were observed in sex distribution across outbreaks (*p* = 0.6953). Age in years, expressed as median and the interquartile ranges, were 43 (26–67) in 2016, 43 (31–59) in 2019 and 42 (26–58) in 2024 (*p* = 0.1000).

### 3.2. DENV Serotyping Results

The infecting DENV serotype was successfully determined in 333 RT-PCR-positive samples, including 82 from the 2016 outbreak, 84 from 2019, and 167 from 2024. These samples correspond to the subset of samples with sufficient viral load (optimal Ct values) to allow serotyping using the CDC protocol. In 2016, positive cases were almost exclusively due to DENV-1 [78 (95.12%)], with a small proportion of DENV-4 [4 (4.88%)]. During 2019, both serotypes circulated again, with DENV-1 remaining predominant [51 (60.71%) were DENV-1 and 33 (39.29%), DENV-4]. In 2024, DENV-2 became predominant [163 (97.60%)], while only a few serotyped samples were attributed to DENV-1 [4 (2.40%)].

### 3.3. Secondary Infection Rates Across Sequential Outbreaks

Based on the classification criteria described in the Methods, 334 of the 869 patients (38.43%) were classified as having secondary dengue infection, whereas 535 (61.57%) had primary infection. The proportion of secondary infections increased progressively across outbreaks, rising from 31.05% (159 of 512) in 2016 to 43.24% in 2019 (64 of 148), to 53.11% (111 of 209) in 2024 (*p* < 0.0001).

### 3.4. Comparison of Clinical Profiles in Primary and Secondary Infections Overall and Stratified by Outbreak

Across the three outbreaks, comparison of clinical parameters between primary and secondary infections revealed that secondary infections had significantly higher hematocrit and hemoglobin levels, lower platelet counts (155,000 vs. 140,000 cells/μL; *p* < 0.0001), and elevated liver enzymes, with higher AST (*p* < 0.0001) and ALT (*p* = 0.0003) (Table 1).

When we compared clinical parameters between primary and secondary DENV infections within each outbreak, secondary infections showed consistently higher levels of hepatic enzymes (AST and ALT) in all three outbreaks. In 2016, secondary infections were also associated with significantly lower platelet counts (*p* = 0.0015) (Supplementary Table S1). In contrast, during 2019, no additional markers of severity were identified when comparing primary and secondary infections (Supplementary Table S1). The most pronounced differences were observed in 2024, where secondary infections exhibited a broader profile of severity-related parameters, including significant changes in hematocrit, hemoglobin concentration, WBC, platelet counts, and elevated hepatic enzymes, AST and ALT (Table 2).

Considering laboratory markers associated with dengue severity, including WBC count < 5000/μL, platelet count < 100,000/μL, and ALT or AST levels above the upper reference limit (40 U/L), we evaluated the proportion of patients presenting at least one abnormal parameter during each outbreak. In 2024, a significantly higher proportion of individuals with secondary infections exhibited at least one abnormal laboratory finding (93.6%) compared with those in 2016 (85.2%) and 2019 (79.2%), indicating a more pronounced severity profile during the most recent outbreak (*p* = 0.0300). Among individuals with secondary infections, 88.3% had WBC counts below 5000/μL, and nearly 80% presented elevated AST levels.

Because age has been associated with dengue severity in previous studies, we further evaluated its potential influence in our cohort. Age was analyzed both as a continuous variable and after stratification into five predefined age groups (1–18, 19–30, 31–45, 46–60, and >60 years). Neither approach showed a significant association with the presence of laboratory severity markers. Mean age did not differ between patients with and without severity markers (46.0 vs. 43.5 years, *p* = 0.37), and no significant association was observed across age categories (χ^2^ = 4.36, df = 4, *p* = 0.36).

### 3.5. Impact of Secondary Infections in Diagnosis

A total of 318 paired samples were analyzed to assess the concordance between NS1 Ag detection and PCR DENV-RNA testing and the ability of the methodologies to detect acute infections during outbreaks. DENV was detected by any of the 2 methodologies in 211 of 318 samples (acute-phase dengue cases were 66.4% overall). A substantial number of samples were detected by a single method only, 56 of 211; 26.5%. Considering RT-PCR as the reference standard, the NS1 RDT showed a sensitivity of 85.6% and a specificity of 78.1%. The positive predictive value was 83.8% and the negative predictive value, 80.5%, resulting in overall accuracy of 82.4%.

We observed that secondary infections were associated with a twofold higher risk of discordant results between NS1 Ag and PCR compared with non-secondary infections (including primary infections and RT-PCR/NS1-negative samples) (RR 2.01; 95% CI 1.23–3.30; *p* = 0.0069) (Table 3). Cohen’s kappa coefficient was 0.640 (κ1), consistent with a moderate-to-high level of agreement between the two diagnostic methods (Table 4). However, agreement between NS1 Ag-RDT and RT-PCR was significantly higher, κ2= 0.759, when considering only the group of non-secondary infections (primary plus RT-PCR/NS1-negative samples) (Table 5) than in secondary infections, κ3 = 0.457 (Table 6). The difference in kappa was statistically significant (κ3 = 0.457 vs. κ2 = 0.759; Δκ = 0.302; Z = 3.22; *p* = 0.0013), indicating reduced concordance in the context of secondary dengue infection.

When the antibody detection platforms of the RDT were compared with ELISA methodologies, the proportion of positive samples identified by the SD BIOLINE IgM and IgG assay was significantly lower than that detected by the ELISA (DIA.PRO) for both markers (*p* < 0.0001), indicating reduced sensitivity of the rapid test. Agreement between the two methods was moderate for IgM and fair for IgG (for IgM: κ = 0.495 SE = 0.054, IC95% = 0.389–0.602; and for IgG: κ = 0.183, SE = 0.053, IC95% = 0.078–0.287). Because of this low overall concordance, analyses by primary versus secondary infection were not considered informative.

## 4. Discussion

This study shows a progressive increase in secondary DENV infections in Posadas, Misiones, across consecutive outbreaks, accompanied by marked shifts in the dominant circulating serotype. Our results suggest that the accumulation of previously exposed individuals, along with the introduction of a new predominant serotype, appeared to contribute to a higher frequency of laboratory abnormalities consistently associated with more severe clinical presentation and reduced diagnostic reliability.

In agreement with national reports [3,4], the 2016 outbreak was almost exclusively caused by DENV-1, with only minimal circulation of DENV-4. By 2019, although DENV-1 remained predominant, DENV-4 accounted for nearly 40% of cases, indicating greater serotype diversification [5,6]. However, routine surveillance in Argentina relies on serotyping a limited number of samples [3,4,5,6,7]. In contrast, our study includes a substantially larger number of serotyped cases, providing a more robust and region-specific characterization of viral circulation. In 2024, the epidemiological scenario changed markedly with the introduction of DENV-2, which rapidly became the dominant serotype among symptomatic infections, representing almost 98% of cases [7,8].

Such serotype shifts are recognized as important drivers of epidemic re-emergence and disease severity [24,25]. Longitudinal cohort studies have shown that most primary dengue infections are inapparent, allowing the silent accumulation of immunity within endemic populations [26]. Repeated outbreaks therefore increase the proportion of individuals with pre-existing immunity, creating favorable conditions for secondary infections when a new serotype is introduced. Supporting this concept, a recent meta-analysis showed that prior dengue infection significantly increased the risk of severe dengue during heterologous secondary infections, despite reducing the likelihood of virologically confirmed infection [27].

Previous studies have demonstrated that secondary infections, particularly those involving DENV-2 or DENV-3 after prior exposure to a different serotype, are more frequently associated with severe outcomes, whereas DENV-1 and DENV-4 infections tend to be milder [28,29]. In our cohort, approximately half of individuals infected with DENV-2 during the 2024 outbreak had secondary infections. The progressive accumulation of secondary infections, together with the emergence of DENV-2 as the predominant circulating serotype in 2024, was associated with a higher frequency of laboratory markers of dengue severity. Sequencing performed subsequently identified the 2024 viruses as DENV-2 genotype II (Cosmopolitan) lineage F.1.1.2. (manuscript in preparation). Although viral genetic variation may contribute to differences in clinical outcome, the effect of this lineage on dengue severity has not yet been established. Therefore, the relative contribution of viral genetics and host immune status to the increased frequency of laboratory severity markers observed in 2024 cannot be determined from the present study.

Although age has been reported as a determinant of dengue severity, we did not observe an association between age and laboratory severity markers in our cohort.

Laboratory abnormalities evaluated in this study have been consistently associated with more severe clinical disease. However, they should not be interpreted as direct evidence of progression to severe dengue as we were unable to determine whether these patients subsequently progressed to clinically severe dengue.

Furthermore, secondary infections alter viral kinetics and antigen availability. Rapid viral clearance driven by pre-existing cross-reactive antibodies and memory immune responses may shorten the diagnostic window for RNA detection while also altering the kinetics and availability of circulating NS1 antigen. Previous studies showed lower median viremia levels in secondary infections compared with primary infections [30,31,32]. Also, early immune complexes formation may mask NS1 epitopes targeted by diagnostic assays, reducing test sensitivity despite ongoing infection [31,33,34].

In our study, secondary infections were associated with reduced concordance between NS1 Ag detection and DENV RNA by RT-PCR, making acute DENV diagnosis more challenging in this immunological context. As a result, a proportion of acute infections during outbreaks may remain undetected depending on the diagnostic methodology used.

Taken together, our findings suggest that the combined use of RT-PCR and NS1 Ag-RDT, along with anti-DENV IgM determination, may be necessary to improve detection of acute DENV infections, particularly in settings with a high frequency of secondary infections. Relying on a single diagnostic approach may underestimate the true number of cases, whereas the complementary use of both methods improves overall diagnostic sensitivity.

### Study Limitations

Despite the strengths of our study, several limitations should be acknowledged. First, although we analyzed a large number of samples across multiple outbreaks within the same population in Posadas, sequential specimens from individual patients were not available, precluding a detailed evaluation of the temporal kinetics of viremia and NS1 antigen levels. Second, the study was based on routine laboratory records, and detailed clinical information and systematic follow-up were unavailable. Consequently, analyses were restricted to objective laboratory markers and hospitalization status, and we were unable to determine whether patients with laboratory abnormalities subsequently progressed to clinically severe dengue. Future prospective studies integrating laboratory findings with longitudinal clinical outcomes will be essential to establish the prognostic significance of these observations. Third, comparisons between primary and secondary infections were restricted to acute-phase samples, which may have resulted in the underrepresentation of secondary infections diagnosed outside this time window. Fourth, information on the duration of residence in the study area was unavailable. Although this variable could provide complementary context regarding cumulative exposure, its absence is unlikely to have affected the main conclusions. Fifth, we were unable to determine the serotype responsible for the primary infection at the individual level. However, national surveillance records and our own data indicate that DENV-1 predominated during earlier outbreaks in the region, suggesting that most secondary infections likely occurred in individuals previously exposed to DENV-1.

## 5. Conclusions

Consecutive outbreaks in Misiones revealed serotype replacement, DENV-2 emergence, and a rising burden of secondary infections associated with an increased frequency of laboratory markers associated with dengue severity and reduced NS1/PCR concordance. Sequential outbreaks progressively reshape population immunity, influencing both disease expression and the performance of routine diagnostic assays.

## Figures and Tables

**Table 1:** Clinical parameters in primary versus secondary infections in the entire population.

Clinical Parameters N = 869	Primary Infections N = 535	Secondary Infections N = 334	*p*
Hematocrit (%)	41.9 (38.8–44.8)	42.7 (39.7–46.0)	**0.0380**
Hemoglobin (g/dL)	14.0 (12.9–15.1)	14.3 (13.3–15.5)	**0.0230**
WBC (cells/μL)	3810 (2960–5296)	3739 (2795–5170)	0.3200
Platelets (cells/μL)	155,000 (131,000–194,000)	140,000 (108,000–176,000)	**<0.0001**
Total protein (g/dL)	7.2 (6.9–7.5)	7.1 (6.8–7.4)	0.3100
Albumin (g/dL)	4.3 (4.0–4.5)	4.2 (4.0–4.4)	**0.0471**
AST (U/L)	36 (26–50)	50 (34–74)	**<0.0001**
ALT (U/L)	28 (20–48)	38 (25–67)	**0.0003**

WBC: white blood cells, ALT: Alanine aminotransferase, AST: Aspartate aminotransferase. Values are presented as medians with the interquartile range in parentheses. Continuous variables were compared using Mann–Whitney test. Confidence intervals were set at 95% (CI95) and *p* value < 0.05 (shown in bold) were considered statistically significant.

**Table 2:** Primary versus secondary in 2024 outbreak.

Clinical Parameters	Primary Infections N = 98	Secondary Infections N = 111	*p*
Hematocrit (%)	42.1 (38.8–44.9)	43.8 (41.1–46.2)	**0.0124**
Hemoglobin (g/dL)	14.1 (13.2–15)	14.8 (13.8–15.7)	**0.0077**
WBC (cells/μL)	3350 (2610–4430)	2880 (2440–3540)	**0.0213**
Platelets (cells/μL)	170,000 (133,500–209,500)	140,000 (119,000–176,000)	**0.0029**
Total protein (g/dL)	7.4 (7.0–7.7)	7.4 (7.1–7.8)	0.7000
Albumin (g/dL)	4.5 (4.3–4.7)	4.4 (4.2–4.8)	0.6000
AST (U/L)	38 (29–50)	50 (40–74)	**0.0007**
ALT (U/L)	27 (19–38)	37 (23–59)	**0.0126**

WBC: white blood cells, ALT: Alanine aminotransferase, AST: Aspartate aminotransferase. Each value is presented as median with the interquartile range in parentheses. Continuous variables were compared using Mann–Whitney test. Confidence intervals were set at 95% (CI95) and a *p* value < 0.05 (shown in bold) were considered statistically significant.

**Table 3:** Frequency of NS1 Ag and DENV RNA discordant results with and without secondary infections.

	Total *	Non-Secondary Infections (Primary Infections Plus RT-PCR/NS1-Negative Samples)	%	Secondary Infections	%
Concordant results	262	153	58.4	109	41.6
Discordant results	56	21	37.5	35	62.5

* N = 318; *p* = 0.0069, RR = 2.01.

**Table 4:** Calculation of Kappa index of agreement between RNA and NS1 Ag in total samples.

*n* = 318	RNA (+)	RNA (-)
NS1 Ag (+)	155	30
NS1 Ag (-)	26	107

κ1 = 0.640; IC95% = 0.554–0.725; SE = 0.044.

**Table 5:** Calculation of Kappa index in samples excluding secondary infections (primary infections plus RT-PCR/NS1-negative samples).

*n* = 174	RNA (+)	RNA (-)
NS1 Ag (+)	77	9
NS1 Ag (-)	12	76

κ2 = 0.759; IC95% = 0.662–0.855; SE = 0.049.

**Table 6:** Calculation of Kappa index in secondary infections.

*n* = 144	RNA (+)	RNA (-)
NS1 Ag (+)	78	21
NS1 Ag (-)	14	31

κ3 = 0.457; IC95% = 0.301–0.614; SE = 0.08.

## Data Availability

The datasets used and/or analyzed during the current study are available from the corresponding author upon reasonable request.

## Supplementary Materials

The following supporting information can be downloaded at https://www.mdpi.com/article/10.3390/v18070785/s1. Table S1: Primary versus secondary in 2016 and 2019 outbreaks.
