# Supplementary material for: Increasing Frequency of Secondary Dengue Infections in Sequential Outbreaks (2016–2024)—Clinical Impact and Diagnostic Challenges
Source: Viruses. 2026 Jul 18;18(7):785. doi: 10.3390/v18070785 (PMC13431616; doi:10.3390/v18070785)
Supplement: Supplementary file 1 [file viruses-18-00785-s001.zip › viruses-4388663-supplementary.pdf]

<u>Supplementary Table S1:</u> *Primary versus secondary in 2016 and 2019 outbreaks*

**Clinical parameters 2016**	**Primary infections N=353**	**Secondary infections N=159**	**p**
Hematocrit (%)	41.0 (38.1-44.7)	41.8 (38.6-45.5)	0.2675
Hemoglobin (g/dl)	13.7 (12.7-15.1)	14.0 (13.1-15.5)	0.1772
WBC (cells/µL)	4290 (3033-5828)	4500 (3648-5373)	0.4148
Platelets (cells/µL)	154,000 (126,500-190,250)	127,500 (97,875-169,250)	**0.0015**
Total protein (g/dL)	7.1 (6.1-7.4)	6.9 (6.7-7.3)	0.1549
Albumin (g/dL)	4.19 (4.0-4.4)	4.1(3.8-4.2)	**0.0245**
AST (U/L)	34 (26-50)	45 (33-76)	**0.0030**
ALT (U/L)	29 (22-52)	40 (27-64)	**0.0176**
**Clinical parameters 2019**	**Primary infections N=84**	**Secondary infections N=64**	**p**
Hematocrit (%)	41.9 (39.4-44.7)	42.1 (39.7-44.8)	0.5862
Hemoglobin (g/dl)	14.1 (13.1-15.1)	14.1 (13.3-15.0)	0.9900
WBC (cells/µL)	3929 (3266-5580)	3760 (2901-5640)	0.6154
Platelets (cells/µL)	156,400 (142,500-206,100)	150,000 (112,450-192,500)	0.0667
Total protein (g/dL)	7.1 (6.9–7.6)	6.9 (6.6 -7.2)	**0.0464**
Albumin (g/dL)	4.3 (4.0-4.5)	4. 2 (3.9-4.4)	0.1266
AST (U/L)	30 (22-47)	51 (26-81)	**0.0175**
ALT (U/L)	30 (18-51)	43 (27-90)	**0.0094**

WBC: white blood cells, ALT: Alanine aminotransferase, AST: Aspartate aminotransferase. Each value is presented as median with the interquartile range in parentheses. Continuous variables were compared using Mann-Whitney test. Confidence intervals were set at 95% (CI95) and a p value < 0.05 (shown in bold) were considered statistically significant.
